# Fully automated immunoassay for cholesterol uptake capacity to assess high-density lipoprotein function and cardiovascular disease risk

**DOI:** 10.1038/s41598-023-28953-x

**Published:** 2023-02-02

**Authors:** Katsuhiro Murakami, Amane Harada, Ryuji Toh, Takuya Kubo, Keiko Miwa, Jeeeun Kim, Maria Kiriyama, Takuya Iino, Youichi Nishikawa, Shin-Nosuke Uno, Kohei Akatsuchi, Manabu Nagao, Tatsuro Ishida, Ken-ichi Hirata

**Affiliations:** 1 Central Research Laboratories, Sysmex Corporation, 4-4-4 Takatsukadai, Nishi-Ku, Kobe, 651-2271 Japan; 2 Division of Evidence-Based Laboratory Medicine, Kobe University Graduate School of Medicine, 7-5-1 Kusunoki-Cho, Chuo-Ku, Kobe, 650-0017 Japan; 3 Sysmex R&D Center Americas, Inc., Mundelein, IL 60060 USA; 4 Division of Cardiovascular Medicine, Kobe University Graduate School of Medicine, Kobe, 650-0017 Japan

**Keywords:** Assay systems, Biomarkers

## Abstract

High-density lipoprotein (HDL) cholesterol efflux capacity (CEC), which is a conventional metric of HDL function, has been associated with coronary heart disease risk. However, the CEC assay requires cultured cells and takes several days to perform. We previously established a cell-free assay to evaluate cholesterol uptake capacity (CUC) as a novel measure of HDL functionality and demonstrated its utility in coronary risk stratification. To apply this concept clinically, we developed a rapid and sensitive assay system based on a chemiluminescent magnetic particle immunoassay. The system is fully automated, providing high reproducibility. Measurement of CUC in serum is completed within 20 min per sample without HDL isolation, a notably higher throughput than that of the conventional CEC assay. CUC decreased with myeloperoxidase-mediated oxidation of HDL or in the presence of *N*-ethylmaleimide, an inhibitor of lecithin: cholesterol acyltransferase (LCAT), whereas CUC was enhanced by the addition of recombinant LCAT. Furthermore, CUC correlated with CEC even after being normalized by ApoA1 concentration and was significantly associated with the requirement for revascularization due to the recurrence of coronary lesions. Therefore, our new assay system shows potential for the accurate measurement of CUC in serum and permits assessing cardiovascular health.

## Introduction

High-density lipoprotein (HDL) particles mediate reverse cholesterol transport (RCT) by carrying excess cholesterol from peripheral tissue, such as the arterial wall, to the liver for excretion into bile. Large-scale epidemiologic studies have shown that HDL cholesterol (HDL-C) is inversely associated with the risk of coronary heart disease^1,2^. Even though modulating HDL has not yet been proven to be a valuable therapy for reducing cardiovascular disease, HDL-C remains an important biomarker. However, the notion that circulating HDL particles are antiatherogenic in humans has been contentious. Direct antiatherogenic effects of HDL or its major protein, apolipoprotein A1 (ApoA1), have been shown in preclinical studies. These effects include prevention or regression of lesions in animal models of atherosclerosis after administration of HDL or ApoA1^3–5^. However, clinical trials and genetic studies have presented conflicting evidence, suggesting that interventions or genetic variants that increase HDL-C levels do not necessarily reduce the risk of coronary heart disease^6–8^. This discrepancy has prompted further studies of HDL properties other than static mass-based measures to help understand the mechanisms that link HDL-related pathways to coronary heart disease.

Traditionally, cholesterol efflux capacity (CEC)—the dynamic rate of the initial step in RCT—has been considered a major metric of HDL function^9^. CEC is measured using a cell-based assay in which cultured macrophages are commonly labeled with ^3^H-labeled cholesterol and subsequently exposed to a cholesterol acceptor, typically apolipoprotein B (ApoB)-depleted serum. Recent large cohort studies have demonstrated that CEC associates with both the prevalence and incidence of cardiovascular disease. This association appears to be a better predictor than steady-state circulating HDL-C concentration^10–12^. However, there is no gold standard protocol for measuring CEC in humans because protocols differ by cholesterol label (e.g., radioisotope label or fluorescent label), cell type (e.g., macrophage, monocyte, hepatocyte), and acceptor (e.g., isolated pure HDL, ApoB-depleted plasma/serum)^13^. Thus, a simpler and standardized method to measure HDL functionality is needed for useful clinical application.

Based on our hypothesis that the efficiency of HDL-induced cholesterol efflux from macrophages depends mainly on the capacity of HDL to take up cholesterol, we have recently established a cell-free assay system to evaluate the capacity of HDL to accept additional cholesterol^14,15^. We have called this cholesterol uptake capacity (CUC), which uses fluorescently labeled cholesterol and an ApoA1-specific antibody. CUC correlated positively with CEC and inversely with the requirement for revascularization because of the recurrence of coronary lesions in patients with optimal control of low-density lipoprotein (LDL) cholesterol^14,15^. We have also reported that CUC showed a significant inverse correlation with the incidence of target-lesion revascularization and with lipid accumulation inside stents, suggesting that neoatherosclerosis contributes to the association between CUC and target-lesion revascularization^16^. Furthermore, CUC was inversely related to the lipid-rich plaque burden and the extent of macrophage accumulation; therefore, CUC could be useful for cardiovascular risk stratification^17^.

In the present study, we aimed to apply the concept of CUC to an automated immunoassay system using polyethylene glycol (PEG)-inserted biotin-labeled cholesterol (Bio-PEG3-cholesterol) and alkaline phosphatase (ALP)-conjugated streptavidin. Subsequently, the association with the need for revascularization was evaluated using the assay system.

## Results

### Development of an automated CUC assay

To develop the automated CUC assay based on the chemiluminescence detection principle, we invented the following assay scheme. HDL was (i) diluted, (ii) incubated with labeled cholesterol, and (iii) captured by an ApoA1-specific antibody coated onto microbeads. (iv) After the addition of ALP-conjugated probe and (v) substrate, chemiluminescence was measured as CUC with an automated immunochemistry analyzer, HI-1000^18^ (Supplementary Fig. 1).

As described in “Materials and methods”, we developed an anti-ApoA1 mouse monoclonal antibody (mAb 8E10) that had minimal recognition bias between oxidatively modified and nonoxidized forms of HDL and between lipid-free and lipidated ApoA1. The antibody bound specifically to ApoA1 and not to other apolipoproteins, such as ApoA2, ApoB, or ApoE isoforms (Supplementary Fig. 2a). In addition, this antibody recognized ApoA1 evenly in serum samples because ApoA1 levels measured with this antibody correlated well with serum ApoA1 concentrations, whereas the commercial anti-ApoA1 antibody (mAb 1C5) used in the previous report^14^ did not show such clear correlation (Supplementary Fig. 2b,c). We also synthesized Bio–PEG3–cholesterol to keep the biotin tag accessible to the streptavidin probe even after the cholesterol structure was incorporated into HDL (Supplementary Scheme).

### Dilution linearity and specificity

**Figure 1:**
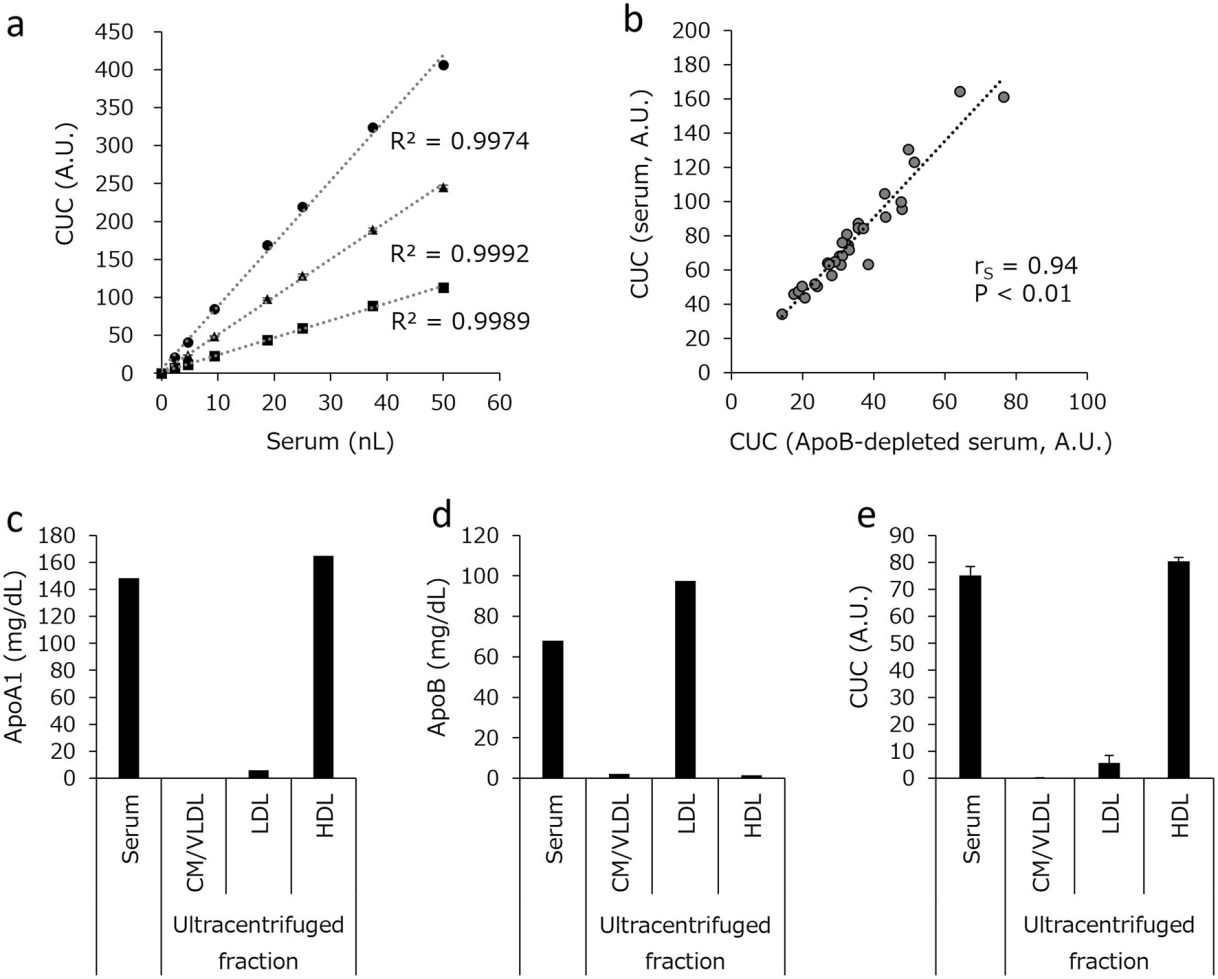
Dilution linearity of CUC and specificity of the automated CUC assays. (**a**) Serial dilutions of pooled serum samples with high (circle), middle (triangle), and low (square) CUC were analyzed. (**b**) CUC of serum and of the corresponding ApoB-depleted serum was measured, and Spearman’s rank correlation coefficient (r_S_) between them was assessed. HDL and other lipoprotein fractions (CM/VLDL, LDL) were extracted from the pooled sera and ApoA1 (**c**), ApoB (**d**), and CUC (**e**) were measured in each fraction. The values in (**a**) and (**e**) represent the mean of triplicate determinations; error bars indicate SD. The values in (**b**–**d**) represent the average of duplicate determinations (CUC) or single determinations (ApoA1 and ApoB). *A.U.* arbitrary units.

The assay was standardized using a pooled serum sample derived from 27 healthy individuals with normal lipid profiles. CUC was determined by interpolation from an unweighted four-parameter standard curve obtained by serially diluted calibrators of the pooled serum. The dilution linearity of the CUC assay system was assessed by measuring five points with different concentrations from the same set of specimens that were sorted into high, middle, and low CUC groups. Specimen dilution analysis results were linear between 0 and 50 nL of serum (R^2^ > 0.99) (Fig. 1a).

To evaluate the specificity of the assay, we first confirmed a good correlation between the CUC of whole serum and that of the corresponding ApoB-depleted serum (Fig. 1b). Therefore, the current assay can directly measure CUC in serum by the establishment of mAb 8E10 and the optimization of reagent conditions without the need of HDL extraction. We also confirmed that CUC measured by our automated system and our previous manual method^14^ showed a good correlation (r_S_ = 0.87, P < 0.01) (Supplementary Fig. 3). Next, the major lipoprotein density classes—chylomicron/very low-density lipoprotein (VLDL), LDL, and HDL—were isolated from the serum sample of a healthy individual. As shown in Fig. 1c,d, the distribution of ApoA1 and ApoB was consistent with the lipoprotein fractions. We also confirmed that the HDL fraction showed much higher CUC values than other lipoprotein fractions (Fig. 1e). Although very low CUC values were detected in LDL, it is likely that large HDL particles were included in the LDL fraction consistent with the detection of ApoA1 in the lipoprotein fraction (Fig. 1c).

### Reproducibility

Control samples with high, middle, and low CUC were used to evaluate the assay for reproducibility during validation trials. The range of the coefficient of variation (CV, %) from the samples prepared from low to high CUC was < 5% (Table 1).

**Table 1:** Assay precision evaluation. H, M, and L indicate control pooled serum specimens with high, middle, and low CUC, respectively. *A.U.* arbitrary units, *SD* standard deviation, *CV* coefficient of variation.

CUC level		H	M	L
n		30	30	30
Mean (A.U.)		176.7	102.9	51.7
Intra-assay	SD	5.4	2.7	1.6
	CV (%)	3.1	2.6	3
Inter-assay	SD	2.9	1.7	1.3
	CV (%)	1.6	1.7	2.5

### Interference

Interference by common blood components was assessed by adding interfering substances to serum samples. As shown in Table 2, the changes between samples with and without typical interfering substances in serum were minor or negligible at physiological concentrations (5% change in CUC values).

**Table 2:** Interference testing with bilirubin, hemoglobin, chyle, and rheumatoid factor.

Interfering substances	Concentration	Mean % difference in CUC
Bilirubin F	201 mg/dL	1.1
Bilirubin C	202 mg/dL	1.1
Hemolytic hemoglobin	4500 mg/dL	− 0.1
Chyle	17700 FTU	5.0
Rheumatoid factor	500 IU/mL	− 0.6

### Spike recovery

The spike recovery was performed using blank serum samples to which were added HDL concentrates of high, middle, or low CUC. The recovery rate of CUC from spiked samples was 94.2–98.4% (Table 3). It was confirmed that the CUC signal was negligible in the blank serum sample (Supplementary Fig. 4).

**Table 3:** Spike recovery testing. HDL concentrates with high, middle, and low CUC were added to blank serum samples and the recovery rate was calculated. *A.U.* arbitrary units.

CUC level	H	M	L
Spike CUC (A.U.)	198	99	49
Recovery (%)	98.4	94.2	96.4

### The steroidal structure of Bio-PEG3-cholesterol is essential for its incorporation into HDL

**Figure 2:**
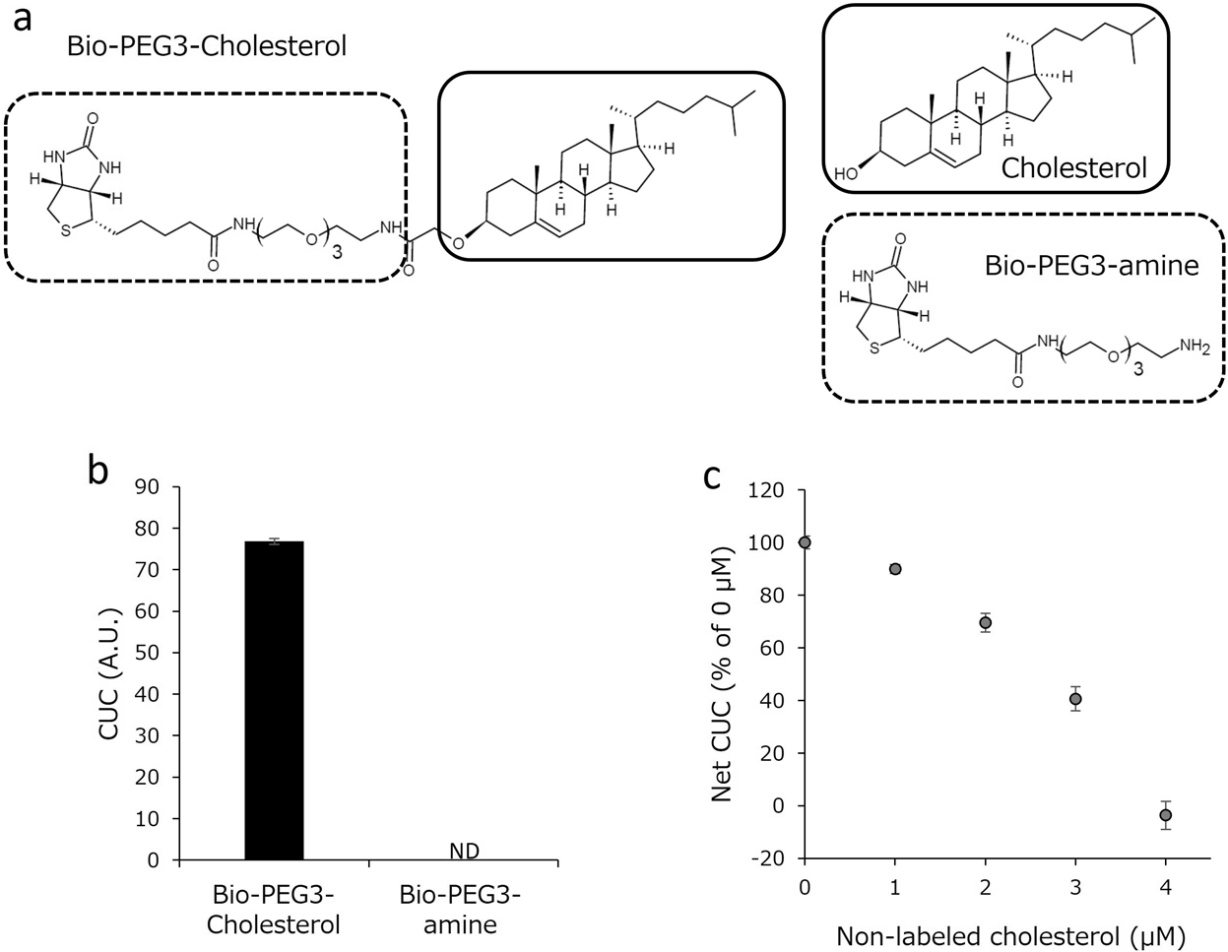
Essential role of the cholesterol structure of Bio-PEG3-cholesterol in its uptake by HDL. (**a**) Structural formulas of Bio-PEG3-cholesterol, free cholesterol, and Bio–PEG3 amine. (**b**) CUC was measured with serum in the presence or absence of Bio-PEG3 amine. (**c**) Serum was incubated with 0.5 μmol/L Bio-PEG3-cholesterol in the presence of an increasing concentration of cholesterol, then CUC was measured with or without pooled serum sample. Net CUC values were calculated by subtracting the background from the total count. All values represent the mean of triplicate determinations; error bars indicate SD. *A.U.* arbitrary units.

The newly designed labeled cholesterol, Bio-PEG3-cholesterol, is composed of cholesterol and a Bio-PEG3 moiety (Fig. 2a). To verify that the incorporation of Bio-PEG3-cholesterol into HDL is dependent on its cholesterol moiety, and not on the label moiety, we examined whether the Bio-PEG3 moiety alone could be incorporated into HDL. It was confirmed that incubation of the Bio-PEG3 moiety with serum produced no signal (Fig. 2b). A competition assay using label-free cholesterol was also performed. As indicated in Fig. 2c, incorporation of the labeled cholesterol into HDL was suppressed by the addition of label-free cholesterol in a concentration-dependent manner. Therefore, we conclude that Bio-PEG3-cholesterol is incorporated into HDL through its cholesterol moiety, as is endogenous free cholesterol.

### CUC reflects the oxidative inactivation of HDL and the lecithin: cholesterol acyltransferase activity

**Figure 3:**
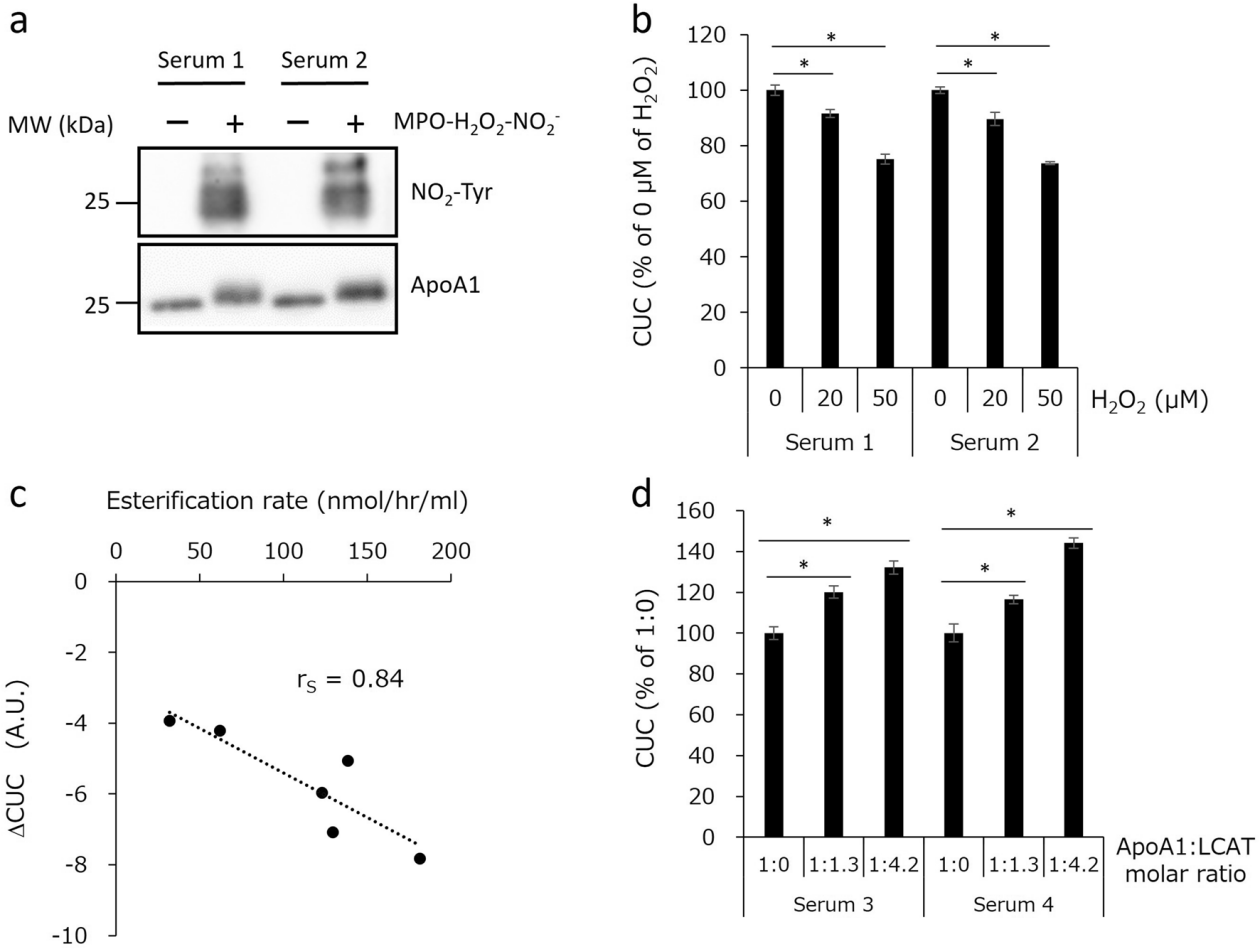
Impairment of CUC by the MPO-mediated oxidation of HDL or in the presence of LCAT inhibitor and its enhancement by the addition of recombinant LCAT. (**a**) Serum samples were oxidized with MPO–H_2_O_2_–NO_2_^−^ and analyzed by western blotting. Original uncropped western blot images are shown in Supplementary Fig. 5. (**b**) CUC of serum modified by MPO–H_2_O_2_–NO_2_^−^. (**c**) CUC of serum samples in the presence or absence of NEM, and the difference between them was calculated as ΔCUC. Basal LCAT activities (esterification rate) of the same set of samples were also measured and compared with ΔCUC. (**d**) CUC of serum samples with low LCAT activity was assessed with or without recombinant LCAT protein. All values represent the means of triplicate determinations; error bars indicate SD. The Mann–Whitney *U* test was used to assess the statistical differences. Asterisks (*) indicate P values less than 0.05 (P < 0.05). *A.U.* arbitrary units.

It has been reported that in vitro oxidation of HDL impairs its ability to promote cholesterol efflux from macrophages^19,20^. In a previous study, we showed that the CUC assay can be used to evaluate the oxidation-induced inactivation of HDL^14^. Therefore, we examined whether the automated CUC assay could reflect the myeloperoxidase (MPO)-mediated inactivation of HDL. First, we exposed serum samples to the MPO-hydrogen peroxide (H_2_O_2_)-nitrite system where western blot analysis confirmed the presence of nitrotyrosine-containing proteins with a molecular weight of about 25 kDa, equivalent to that of ApoA1 (Fig. 3a). We then performed CUC assay using these samples and found that CUC in serum samples was suppressed by MPO-mediated oxidation (Fig. 3b). These results indicate that the automated CUC assay can be used to evaluate oxidation-induced HDL inactivation.

In the process of RCT, cholesterol may be esterified by the enzyme lecithin: cholesterol acyltransferase (LCAT) and transferred to the core of the HDL particle^21^. According to a previous report, ApoA1 in atherosclerotic plaque-laden aorta is markedly functionally impaired with respect to both cholesterol acceptor and LCAT activities^22^. Therefore, we hypothesized that LCAT activity might affect the effectiveness of cholesterol uptake by HDL. To verify this hypothesis, we measured basal LCAT activities and CUC of serum samples in the presence and absence of *N*-ethylmaleimide (NEM), which is an LCAT inhibitor, and calculated the difference between CUC levels of NEM-inhibited and NEM-free samples (ΔCUC). As shown in Fig. 3c, there was a clear inverse correlation between LCAT activity and ΔCUC. We also observed that the addition of recombinant LCAT enhanced the CUC of samples with low LCAT activity (Fig. 3d). It should be noted that the labeled cholesterol used in this study (Fig. 2a) does not contain the hydroxyl group of cholesterol, which is essential for LCAT-mediated esterification^21^. However, we speculate that the space produced by the esterification of endogenous free cholesterol on the surface of HDL can accommodate additional (labeled) cholesterol. Consequently, LCAT activity positively affects the efficiency of cholesterol uptake; thus, the CUC assay may reflect LCAT activity.

### CUC correlates qualitatively with CEC

**Figure 4:**
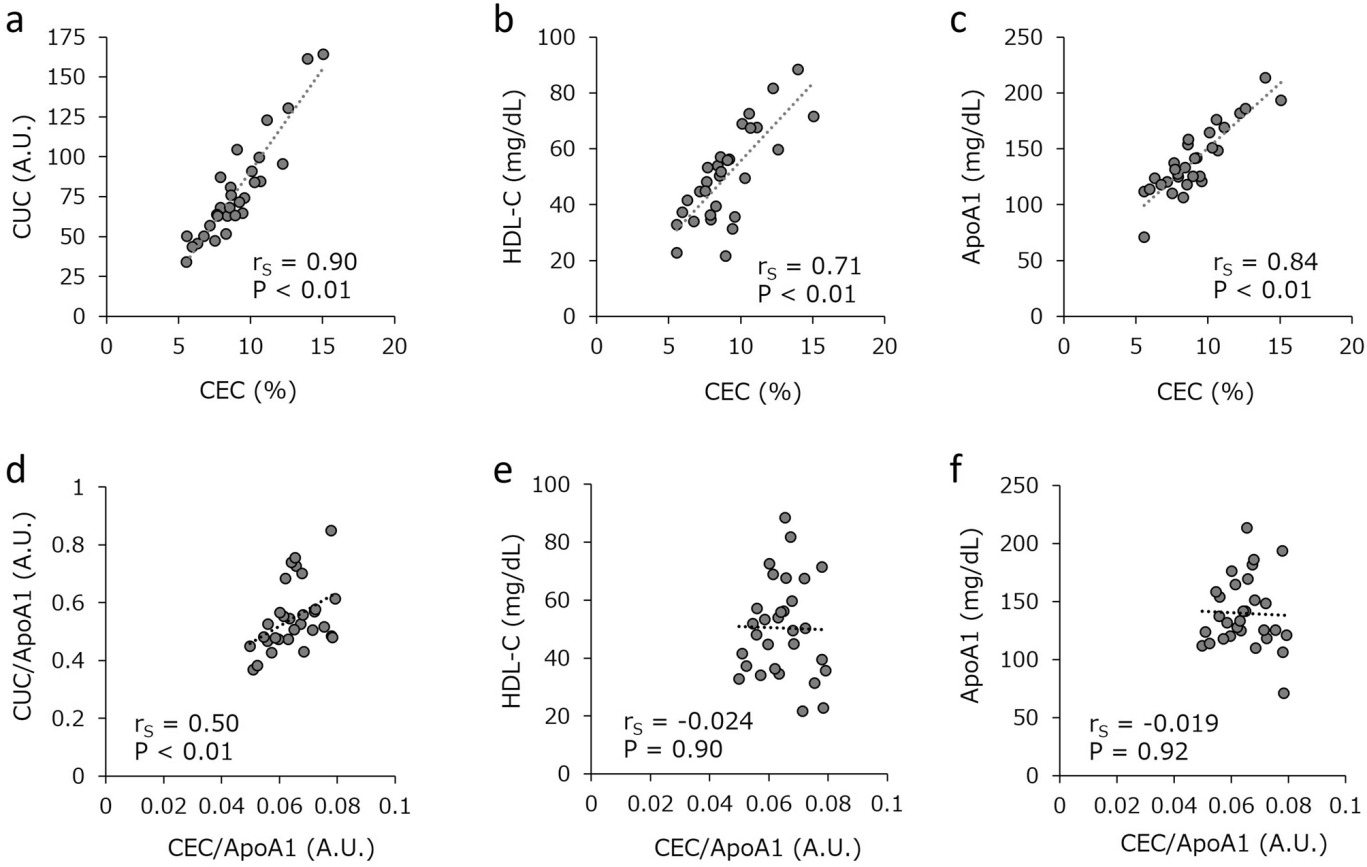
Relationship between CUC and CEC. Spearman’s rank correlation coefficient (r_S_) between CEC and CUC (**a**), serum HDL-C (**b**), or serum ApoA1 (**c**) were analyzed. The r_S_ coefficients between CEC and CUC normalized by serum ApoA1 (**d**), serum HDL-C (**e**), or serum ApoA1 (**f**) were also assessed. Serum and ApoB-depleted serum from 30 individuals were evaluated. The values represent single determinations (serum HDL-C and ApoA1) or the average of duplicate determinations per individual (CEC, CUC, CEC/ApoA1, and CUC/ApoA1). *A.U.* arbitrary units.

The objective of this study was to develop an automated assay system that was suitable for use in clinical settings to assess HDL functionality in RCT. Therefore, we analyzed the association between CEC and CUC measured by the current automated system. As shown in Fig. 4a, BODIPY-labeled-cholesterol efflux from J774 cells correlated significantly with Bio–PEG3–cholesterol uptake (r_S_ = 0.90, P < 0.01), but it also showed good correlation with the metrics of HDL quantity, serum HDL-C (r_S_ = 0.71, P < 0.01) and ApoA1 concentration (r_S_ = 0.84, P < 0.01) (Fig. 4b,c). To compare CEC and CUC more qualitatively, the association between CEC and CUC normalized by ApoA1 was also assessed. Figure 4d shows that a significant association remained between the two values (r_S_ = 0.50, P < 0.01), whereas no such relationship was observed between the normalized CEC and either serum HDL-C or ApoA1 (Fig. 4e,f). These findings suggest that CUC measured by this system correlates with CEC quantitatively and qualitatively, and so can be used as a functional measure for HDL as well as CEC.

### CUC associates with the risk of coronary artery disease (CAD) recurrence

Finally, we examined the performance of CUC in the risk stratification of patients with CAD. The clinical characteristics of the participants showed that 28% of 206 sequential consenting subjects who had previously undergone successful revascularization required revascularization because of recurrent coronary lesions (Supplementary Table 1). Patients in the revascularization group had a significantly higher incidence of diabetes mellitus (DM) (56.9% vs 37.8%; P = 0.01). The recurrence of coronary lesions was inversely associated with CUC (84.5 [73.8–98.1] vs 92.3 [75.9–109.2], P = 0.03), while there was no significant difference in serum HDL-C and ApoA1 levels between the revascularization and nonrevascularization groups (Supplementary Table 1).

A multivariate logistic regression model was used to investigate the factors associated with subsequent revascularization. We found a significant association between CUC and subsequent revascularization (odds ratio 0.41; 95% confidence interval 0.17–0.99, P = 0.047) independent of other variables including HDL-C and DM (Table 4). Therefore, CUC measured by the fully automated assay system could be used to evaluate the recurrence risk of CAD.

**Table 4:** Uni- and multivariate logistic regression analysis for revascularization. *CI* confidence interval, *CUC* cholesterol uptake capacity, *HDL-C* high-density lipoprotein cholesterol, *LDL-C* low-density lipoprotein cholesterol, *OR* odds ratio, *TG* triglyceride.

Variables	Univariate			Multivariate		
	OR	95%CI	*p*-value	OR	95%CI	*p*-value
Age	0.84	0.62–1.13	0.25			
Male sex	1.59	0.68–3.71	0.28			
Current smoking	1.14	0.54–2.37	0.73			
Hypertension	0.78	0.39–1.56	0.49			
Diabetes mellitus	2.17	1.17–4.02	0.01			
TG	1.20	0.88–1.65	0.25			
HDL-C	0.80	0.58–1.11	0.18			
LDL-C	1.21	0.89–1.63	0.22			
CUC	0.68	0.49–0.95	0.02	0.41	0.17–0.99	0.047

## Discussion

In a previous report, we constructed a manual microplate-based assay for measuring CUC as a novel indicator of HDL functionality^14^. Although the manual CUC assay is simpler and affords higher throughput than the conventional, cell-based CEC assay, the manual CUC procedure requires the handling of multiple reagents with precise timing to ensure experimental accuracy and reproducibility. Thus, we used the automated immunochemistry analyzer HI-1000, which is based on chemiluminescent magnetic particle immunoassay technology, to develop an assay system with good precision, reproducibility, and high sensitivity, for the direct measurement of CUC without HDL isolation from serum.

HI-1000 is an immunoassay system for research use based on the HISCL™ system, which is widely used for in vitro diagnostics in several diseases, such as infectious diseases, cancers, and cardiovascular diseases^23–28^. The main advantages of our automated immunoassay system over microplate-based assays are as follows: (1) because of the liquid–liquid reaction, the measurement can be completed with a short turnaround time (less than 20 min) and with high sensitivity; (2) the larger amounts of antibodies and substrates in liquids provide more capacity for the reaction, expanding its dynamic range; and (3) all processes including sample dilution are fully automated, resulting in high reproducibility. These three conditions are essential for clinical use, especially considering the necessity of sharing cutoff values between different facilities.

Lipid-laden macrophages can release cholesterol to HDL through several pathways^29^. ABCA1 is an important player in HDL biogenesis. Conversely, the aqueous diffusion pathway contributes to the maturation of HDL. Because the CUC is determined using a cell-free assay, the CUC appears to reflect the contribution of HDL to cholesterol removal via the aqueous diffusion pathway. However, as shown in the current and previous reports, CUC is significantly associated with CEC and recurrence of CAD^14,16^. These results imply that cholesterol uptake is one of the major steps in the cholesterol efflux pathway and RCT. Recently, it has been shown that a composite proteomic score of HDL that was isolated by recombinant His-tagged ApoA1 protein was highly correlated with CEC and had an inverse association with CAD^30^. Moreover, assays to monitor lipid uptake by plasma HDL from donor lipid particles with fluorescently labeled cholesterol or phospholipid as alternative assays for determining CEC have been developed^31,32^. These reports also support the concept of the cell-free HDL function assay, but further studies are needed to confirm the utility of the CUC assay in the clinical setting.

Because the ultracentrifugation process for HDL isolation takes several days, most of the recent reports have used ApoB-depleted serum as a cholesterol acceptor for the conventional CEC assay. However, manual procedures with multiple steps are necessary to prepare ApoB-depleted serum and therefore this process can increase the risk of mixing samples. In this study, we have established an assay system applicable to the direct measurement of CUC in serum using a specific antibody and optimized reagents without the need for extracting HDL. That is one of the major advantages of this automated antibody-based assay, which can allow the measurement of crude samples without labor-intensive and time-consuming sample preparation.

ApoA1 is the most abundant apolipoprotein contained in HDL, but there are other apolipoproteins in HDL that have been reported to associate with various diseases, such as diabetes, dementia, and cancers^33–35^. These apolipoproteins are also carried by other types of lipoproteins. Moreover, HDL acts as a scavenger of toxic substances including cholesterol, amyloid beta peptides, and oxidized phospholipids^36,37^. Considering these findings, the CUC assay might—by changing the combination of capture antibody and substrate—be applicable to evaluate the functionality of other apolipoprotein-containing HDL or other lipoproteins, and thus be able to diagnose and/or monitor a wide range of diseases.

In summary, we have established a rapid and sensitive assay system to measure the CUC of HDL in crude serum samples as a potential tool for predicting the risk of CAD after percutaneous coronary intervention. The CUC assay can reflect posttranslational modification of HDL and LCAT activity status. This is an incremental step toward demonstrating the broader feasibility of automated HDL function analysis in the clinical laboratory and presents an opportunity to establish the clinical validity and explore the utility of CUC.

## Materials and methods

### Apparatus

The HI-1000 is a fully automated immunoassay system for research applications based on a chemiluminescence enzyme immunoassay methodology that allows highly sensitive measurement with small sample volumes (5–30 μL/test) (Sysmex Corporation, Kobe, Japan). The instrument pipettes samples directly from sample tubes and processes immunoassays and performs data reduction with a steady-state throughput of approximately 30 tests/h in the CUC assay.

### Preparation of ApoB-depleted serum, blank serum, and serum lipoproteins

Serum samples were thawed on ice and incubated with the same volume of 22% PEG 4000, to remove ApoB-containing lipoproteins. In brief, each serum sample was mixed with PEG solution and kept at room temperature for 20 min. The samples were then centrifuged at 860×*g* for 15 min, to precipitate all ApoB-containing lipoproteins, and the supernatant was collected as the ApoB-depleted serum. Blank serum was prepared by depleting ApoA1-containing lipoproteins from pooled serum with anti-ApoA1 antibody (mAb 8E10)-coated microbeads. High, middle, or low CUC controls were prepared from 2, 27, or 4 individual commercial serum samples, and their ApoA1 concentrations were 164.5 mg/dL, 137.0 mg/dL, or 135.8 mg/dL, respectively. Serum lipoproteins were fractionated by ultracentrifugation (Lipo UC, Skylight Biotech, Akita, Japan). ApoA1 and ApoB concentrations in each fraction were measured with N-Assay TIA Apo A1-H Nittobo and N-Assay TIA Apo B-H Nittobo (Nittobo Medical, Tokyo, Japan), respectively. Aliquots of the ApoB-depleted serum and fractioned serum lipoproteins were stored at − 80 °C.

### Generation of mouse monoclonal antibody 8E10

Hybridoma cell lines were generated by immunizing C57BL/6 mice with recombinant human ApoA1 protein (Sigma-Aldrich, St. Louis, MO, USA). Mouse immunization and generation of hybridoma cell lines were outsourced to Cell Engineering Corporation (Osaka, Japan). Hybridoma culture supernatants that contained antibodies with the desired binding specificity for equal recognition of nonoxidized and oxidized HDL were screened using ELISA. In brief, 1 μg/mL of recombinant human ApoA1 protein or ApoB-depleted serum with an ApoA1 concentration of 1 μg/mL, diluted in phosphate-buffered saline (PBS), were immobilized on 96-well plates at 37 °C for 1 h. After washing the wells with PBS, PBS without or with H_2_O_2_ (final concentration 1 mol/L), sodium nitrite (200 μmol/L), and diethylenetriaminepentaacetic acid (DTPA) solution (100 μmol/L) were added to the wells and incubated at 37 °C for 1 h. The wells were washed with PBS and blocked with 2% bovine serum albumin (BSA) in PBS at 25 °C for 1 h. The plates were then incubated with hybridoma culture supernatant at 25 °C for 1 h, followed by the addition of horseradish peroxidase (HRP)-conjugated goat anti-mouse IgG (Dako, Nowy Sącz, Poland) at 25 °C for 30 min. The wells were washed five times with PBS, SuperSignal ELISA pico chemiluminescent substrate (Thermo Fisher Scientific, Waltham, MA, USA) was added to the wells, and the chemiluminescence signal was measured with an Infinite F200 Pro microplate reader (Tecan, Männedorf, Switzerland). mAb 8E10 was selected by screening for equal recognition of lipid-free (recombinant protein) and lipidated (ApoB-depleted serum) ApoA1 under native conditions, as well as after oxidation by exposure to H_2_O_2_-sodium nitrite. To obtain sufficient antibodies for this study, mAb 8E10 was purified from ascites fluid of ICR nude mice using protein A-Sepharose chromatography. Preparation of mouse ascites fluid and purification of mAb 8E10 were outsourced to Kitayama Labes (Nagano, Japan).

All methods were carried out in accordance with relevant guidelines and regulations. All experimental protocols were approved by the Institutional Animal Care and Use Committee at Sysmex Corporation. The study was carried out in compliance with the ARRIVE guidelines.

### Automated CUC assay

The serum sample was diluted in PBS and the diluted sample was incubated with 0.5 μM Bio-PEG3-labeled cholesterol in reaction buffer (PBS containing 0.3% casein Na) at 42 °C for 3 min. HDL was captured by an anti-ApoA1 antibody (mAb 8E10) coated on magnetic particles at 42 °C for 2 min. After washing the particles with wash buffer, 100 μL of alkaline phosphatase-conjugated streptavidin (Promega, Madison, WI, USA) in dilution buffer (0.1 M triethanolamine (pH 7.5) containing 10 mg/mL BSA, 5 mg/mL casein Na, 1 mM MgCl_2_, and 0.1 mM ZnCl_2_) was added and the mixture was incubated at 42 °C for 3 min. After washing the particles, CDP-Star chemiluminescent substrate was added, and the mixture was incubated at 42 °C for 5 min. Chemiluminescence was then measured by count. All procedures were performed with the HI-1000 instrument. The CUC assay was standardized using pooled serum. CUC was also measured in the presence of 6 mM NEM, the LCAT inhibitor. ApoB-depleted serum was incubated with recombinant LCAT protein (Sino Biomedical, Beijing, China) at 37 °C for 1 h before the CUC assay was performed. The LCAT activity of serum was measured with a Roar LCFC LCAT Activity Assay Kit (Roar Biomedical, New York, NY, USA) by following the manufacturer’s instructions.

### Immunoblot analysis

As described in a previous report with minor modifications^14^, serum samples were boiled in sodium dodecyl sulfate–polyacrylamide gel electrophoresis (SDS–PAGE) loading buffer (2% SDS, 10% glycerol, 1 mM dithiothreitol, 0.002% bromophenol blue, 62.5 mM Tris–HCl, pH 6.8), resolved using SDS–PAGE, and transferred onto a polyvinylidene difluoride membrane. The proteins were detected using appropriate primary and HRP-conjugated secondary antibodies, followed by western blotting substrate.

### CEC assay

Macrophage-specific efflux capacity was measured using boron dipyrromethene (BODIPY)–cholesterol by a method reported previously with some modifications^14,38^. J774 mouse macrophages were dispensed into a 48-well plate at 7 × 10^4^ cells per well. After 24 h, the cells were incubated for 2.5 h with 8 μM BODIPY–cholesterol in Dulbecco's Modified Eagle Medium (DMEM) with 10% fetal bovine serum and 10 mM methyl-β-cyclodextrin. After washing with phenol-red-free DMEM, the BODIPY–cholesterol-labeled cells were incubated with 0.5% ApoB-depleted serum in phenol-red-free DMEM for 24 h at 37 °C. The resulting quantity of BODIPY–cholesterol in the medium and the cells was measured with a microplate reader. The percent cholesterol efflux was calculated by the following formula: 100 × fluorescence intensity (FI) of BODIPY–cholesterol in media/(FI of BODIPY–cholesterol in cells + FI of BODIPY–cholesterol in the media).

### MPO-induced oxidation of serum

Serum samples were oxidized by MPO as described in a previous report with minor modifications^14^. Reactions were performed at 37 °C for 1 h in PBS with 100 μmol/L DTPA, pH 7.4, containing serum (16.7 μg/mL ApoA1) supplemented with 100 nmol/L MPO, 200 μmol/L sodium nitrite, and 20 or 50 μmol/L H_2_O_2_. The reaction was started by adding the oxidant and stopped by adding 2 mmol/L methionine.

### Subjects

The serum samples used for the construction and evaluation of the CUC assay were obtained from a commercial source (ProMedDx, Norton, MA, USA). To assess the relationship between CUC and the recurrence of CAD, we enrolled 206 consecutive patients who had previously undergone successful percutaneous coronary intervention or coronary artery bypass grafting and who had been hospitalized for coronary angiography between December 2014 and March 2019 because of simple follow-up, or stable angina, or inducible ischemia. The participants who required coronary revascularization because of restenosis of the original target lesion and/or the occurrence of other nontargeted coronary atherosclerotic lesions were classified as having CAD recurrence^14^. The exclusion criteria were the patients with renal failure (creatinine > 3.0 mg/dL), a history of cancer in the past five years, or missing values in clinical datasets.

The study protocol was in accordance with the ethical guidelines of the 1975 Declaration of Helsinki. The study was approved by the Ethics Review Committee at Kobe University and was registered at the UMIN Clinical Trials Registry (identification number 000034324). Written informed consent was obtained from all patients before enrollment in the study.

### Statistical analysis

Statistical analysis was performed using StatFlex v.7.0 software (Artech, Osaka, Japan) and was expressed as mean, standard deviation (SD), and CV for each test parameter. The Spearman's rank correlation coefficient (r_S_) was calculated to measure the strength and direction of the relationship between two variables. The Mann–Whitney *U* test was used for comparisons between patients with or without recurrence. P values < 0.05 were considered statistically significant.

## Supplementary Information

Supplementary Information.

## Data Availability

The datasets generated and/or analyzed during the current study are available from the corresponding author on reasonable request.

## Abbreviations

ALP: Alkaline phosphatase
ApoA1: Apolipoprotein A1
ApoB: Apolipoprotein B
Bio-PEG3-cholesterol: Polyethylene glycol-inserted biotin-labeled cholesterol
CAD: Coronary artery disease
CEC: Cholesterol efflux capacity
CUC: Cholesterol uptake capacity
HDL: High-density lipoprotein
HDL-C: HDL cholesterol
LCAT: Lecithin: cholesterol acyltransferase
LDL: Low-density lipoprotein
PEG: Polyethylene glycol
RCT: Reverse cholesterol transport
VLDL: Very low-density lipoprotein
