# Supplementary material for: Fully automated immunoassay for cholesterol uptake capacity to assess high-density lipoprotein function and cardiovascular disease risk
Source: Sci Rep. 2023 Feb 2;13:1899. doi: 10.1038/s41598-023-28953-x (PMC9895055; doi:10.1038/s41598-023-28953-x)
Supplement: Supplementary file 1 — Supplementary Information. [file 41598_2023_28953_MOESM1_ESM.pdf]

Supplementary Information

Fully automated immunoassay for cholesterol uptake capacity to assess high-density lipoprotein function and cardiovascular disease risk

Katsuhiro Murakami[1], Amane Harada[1,*], Ryuji Toh[2,*], Takuya Kubo[1], Keiko Miwa[1], Jeeeun Kim[1], Maria Kiriyama[1], Takuya Iino[1], Youichi Nishikawa[1], Shin-Nosuke Uno[1], Kohei Akatsuchi[3], Manabu Nagao[2], Tatsuro Ishida[4] & Ken-ichi Hirata[2, 4]
c

[1] Central Research Laboratories, Sysmex Corporation, Kobe 651-2271, Japan, [2] Division of Evidence-based Laboratory Medicine, Kobe University Graduate School of Medicine, Kobe 650-0017, Japan, [3] Sysmex R&D Center Americas, Inc., Mundelein, IL 60060, USA and [4] Division of Cardiovascular Medicine, Kobe University Graduate School of Medicine, Kobe 650-0017, Japan.

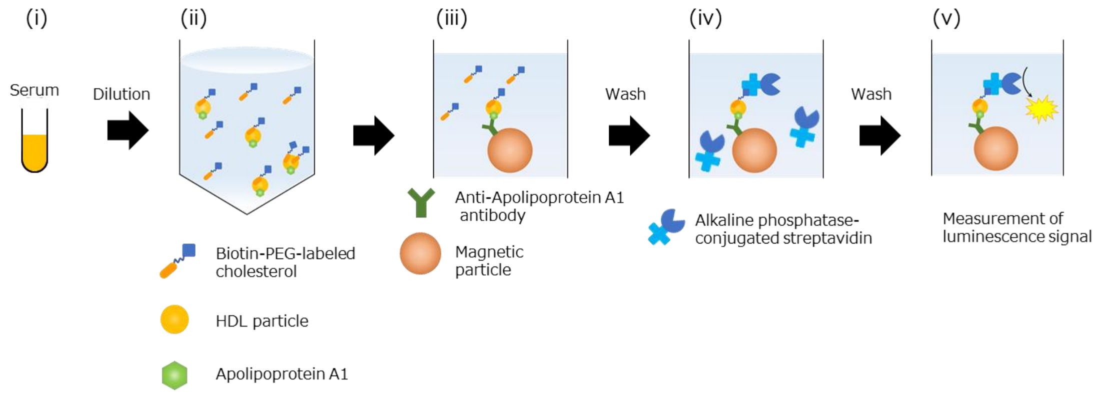

Supplementary Figure 1. Schematic diagram of the fully automated CUC assay. (i) Serum sample is diluted. (ii) The diluted serum is incubated with Bio–PEG3–cholesterol. (iii) HDL with the labeled cholesterol is captured by an anti-ApoA1 antibody coated on microparticles. (iv) After the microparticles are washed, ALP-conjugated streptavidin is added to label the cholesterol. (v) After the microparticles are washed, substrate reagent is added and chemiluminescence is measured. All procedures are processed in the HI-1000 analyzer.

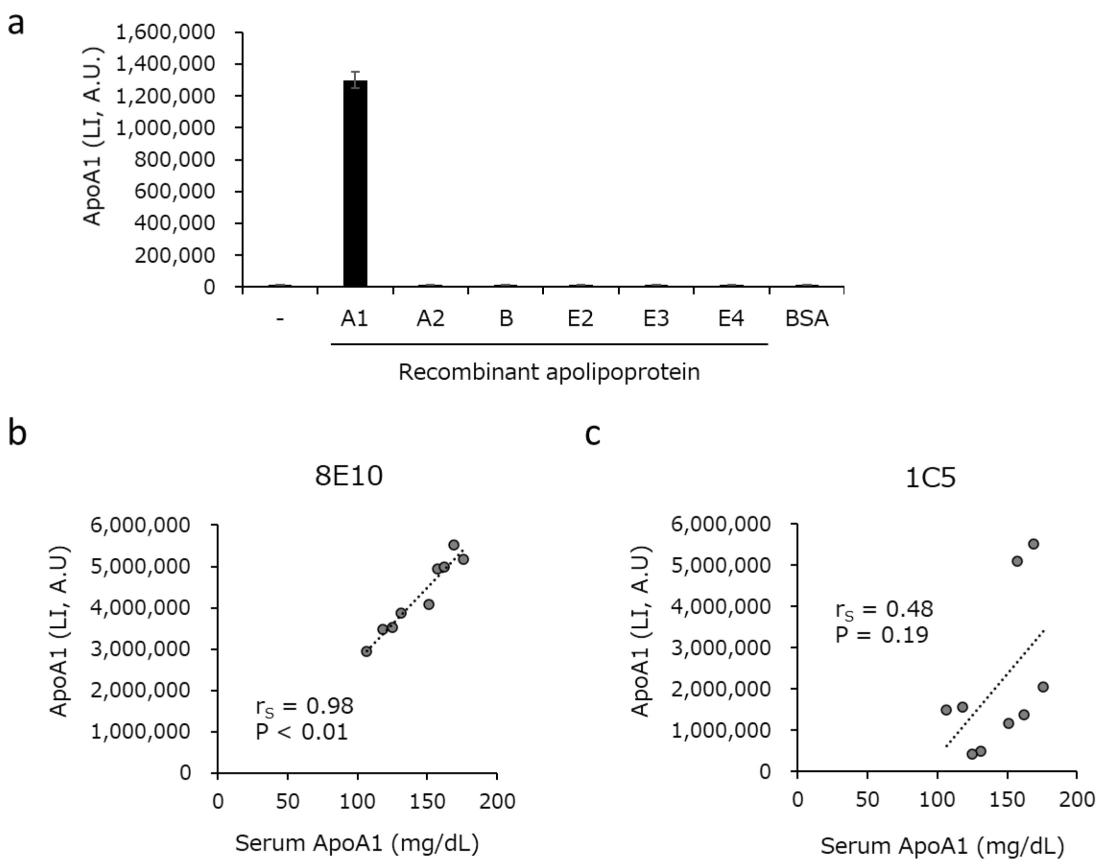

Supplementary Figure 2. Development of a monoclonal anti-human ApoA1 antibody with minimal recognition bias between serum samples. Recombinant apolipoproteins were directly coated onto a microplate and detected with an in-house anti-ApoA1 antibody (mAb 8E10) and HRP-conjugated secondary antibody (**a**). Spearman's rank correlation coefficient (r_S) between serum ApoA1 concentration and ApoA1 levels captured by mAb 8E10 (**b**) or a commercial anti-ApoA1 antibody (mAb 1C5) (**c**). Both were detected by anti-human ApoA1 antisera which is the reagent for the biochemical measurement of serum ApoA1 and HRP-conjugated secondary antibody. The values in **a** represent the means of triplicate determinations; error bars indicate SD. The values in **b** and **C** represent the means of duplicate determinations. LI, luminescence intensity; A.U., arbitrary units.

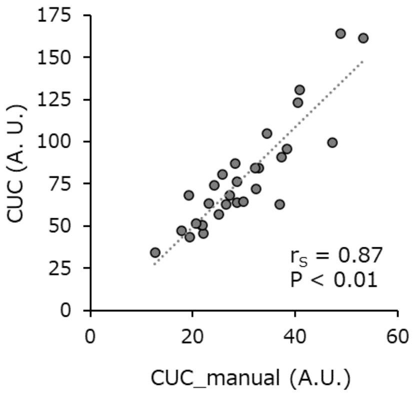

Supplementary Figure 3. Comparison between the CUC of the automated system and the manual method [14] by Spearman's rank correlation coefficient (r_S) between them. Serum and ApoB-depleted serum from 30 individuals were evaluated. The values represent the means of duplicate determinations.

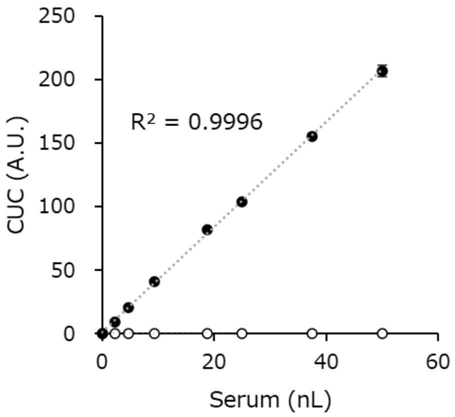

Supplementary Figure 4. Analysis of blank serum sample. CUC was measured in serial dilutions of pooled serum (closed circle) and ApoA1-depleted sample of the same pooled serum (open circle). Serum from 10 individuals was evaluated. The values represent the means of triplicate determinations; error bars indicate SD.

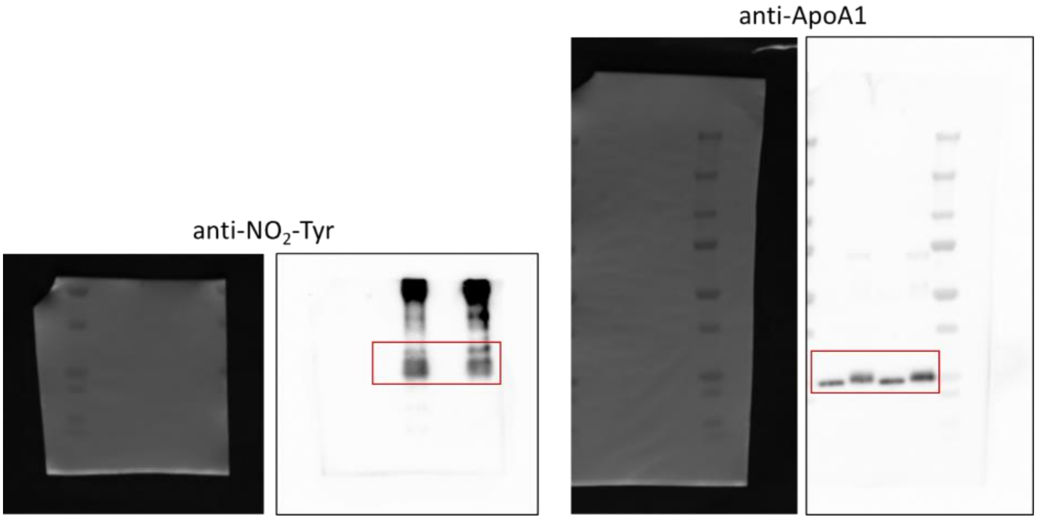

Supplementary Figure 5. Full unedited western blots. The cropped area corresponding to Fig. 3a in the manuscript is shown by a red frame.

Supplementary Table 1. Characteristics of the study subjects.

	Revascularization (n=58)	Non-revascularization (n=148)	*p*-value
Age (yr)	69.3 ±10.9	71.1 ±9.7	0.25
Male, n (%)	50 (86.2)	118 (79.7)	0.28
Body mass index (kg/mm^2)	25.2 ±4.2	24.6 ±4	0.37
Current smoking, n (%)	13 (22.4)	30 (20.3)	0.73
Family history of CAD, n (%)	9 (15.5)	12 (8.1)	0.11
Hypertension, n (%)	42 (72.4)	114 (77)	0.49
Dyslipidemia, n (%)	49 (84.5)	129 (87.2)	0.61
Diabetes mellitus, n (%)	33 (56.9)	56 (37.8)	0.01
Lipid-lowering agent, n (%)	55 (94.8)	140 (94.6)	0.95

Statin use, n (%)	53 (91.4)	137 (92.6)	0.77
Ezetimibe use, n (%)	10 (17.2)	15 (10.1)	0.16
PCSK9 inhibitor use, n (%)	0 (0)	5 (3.4)	0.16
EPA use, n (%)	10 (17.2)	22 (14.9)	0.67
Fibrate use, n (%)	1 (1.7)	3 (2)	0.89
Laboratory data			
Triglyceride (mg/dL)	132.5 (85-161)	115 (80-156.5)	0.65
Total cholesterol (mg/dL)	144 (121-165)	142.5 (124-161)	0.76
HDL cholesterol (mg/dL)	44 (36-51)	44 (38-52.5)	0.46
LDL cholesterol (mg/dL)	77 (66-97)	78 (58-94.5)	0.41
HbA1c (NGSP) (%)	6.3 (5.9-7.1)	6.2 (5.8-6.8)	0.31
eGFR (ml/min/1.73m2)	64.4 (54.8-74.5)	62.5 (52.4-70.6)	0.37
High-sensitivity CRP (mg/dL)	0.04 (0.02-0.11)	0.04 (0.02-0.11)	0.71
BNP (pg/dL)	34.5 (20.9-69.2)	33.5 (16.3-73.3)	0.8
ApoA1 (mg/dL)	117.5 (106-127)	120.5 (106.5-133)	0.18
CUC (A.U.)	84.5 (73.8-98.1)	92.3 (75.9-109.2)	0.03

Abbreviations: BNP, brain natriuretic peptide; CAD, coronary artery disease; CRP, C-reactive protein; CUC, cholesterol uptake capacity; eGFR, estimated glomerular filtration rate; EPA, eicosapentaenoic acid; HbA1c, hemoglobin A1c; HDL, high-density lipoprotein; LDL, low-density lipoprotein; A.U., arbitrary units.

Synthesis of Bio–PEG3–cholesterol

Materials and general information

General chemicals were purchased from commercial suppliers (Fujifilm Wako Pure

Chemical (Osaka, Japan), Tokyo Chemical Industries (Tokyo, Japan), and Sigma-Aldrich Japan (Tokyo, Japan)) and used without further purification. ^{1}H nuclear magnetic resonance (NMR) spectra were recorded on a JEOL JNM-AL400 with chemical shifts (δ) reported in ppm relative to tetramethylsilane (δ = 0.0 ppm), and coupling constants were reported in hertz (Hz). The abbreviations for splitting patterns are as follows; s: singlet, d: doublet, t: triplet, m: multiplet, br.s.: broad singlet. Mass spectra (MS) were measured on a Waters UPLC system with an SQ Detector 2. HPLC purification was performed on a reverse-phase column (Inertsil ODS-3 10 mm × 250 mm; GL Sciences (Tokyo, Japan)) using a Shimadzu (Tokyo, Japan) HPLC system (Prominence) with an LC-20AR pump and an SPD-M40 PDA detector.

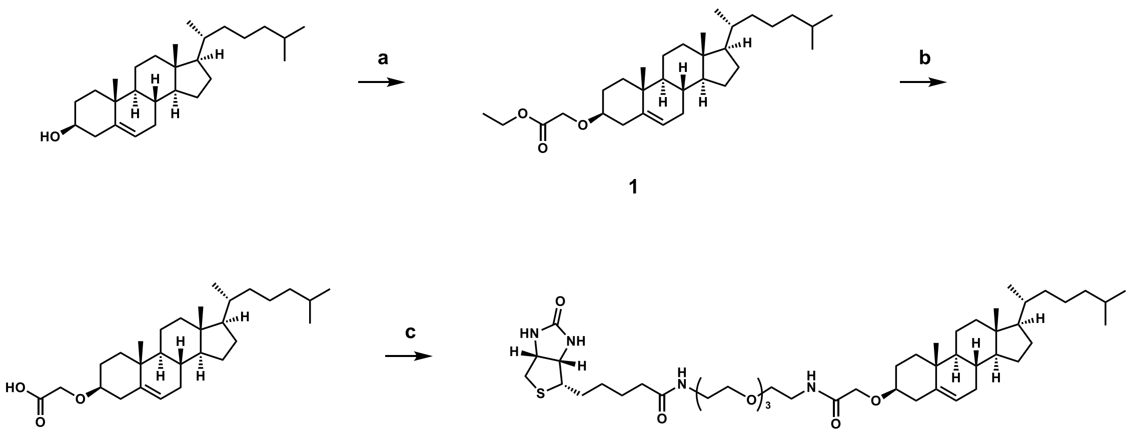

Bio-PEG3-cholesterol

Supplementary Scheme. Synthetic route of Bio–PEG3–cholesterol: (a) Ethyl bromoacetate, NaH, THF, 0 °C to r.t., 18 h; (b) KOH, THF/H_2O, r.t., 1 h; (c) Biotin–PEG3–NH_2, HATU, TEA, DMF, r.t., overnight.

Synthesis of **1**

To a solution of cholesterol (1.16 g, 3 mmol) in THF (15 mL) was added sodium hydride (NaH) (432 mg, 9 mmol) at 0 °C. After stirring at 0 °C for 20 min, ethyl bromoacetate (664 μL, 6 mmol) was added. After further stirring at r.t. for 18 h, water was added, and the mixture was extracted with ethyl acetate. The organic layer was washed with brine, dried over sodium sulfate, filtered, and evaporated. The residue was purified by flash silica gel column chromatography (*n*-hexane:ethyl acetate 15:1) to give **1** as a white solid (182 mg, 13% yield). ESI-MS (m/z): calcd. for $C_{31}H_{52}NaO_3$, 495; found 495, $[M+Na]^+$.

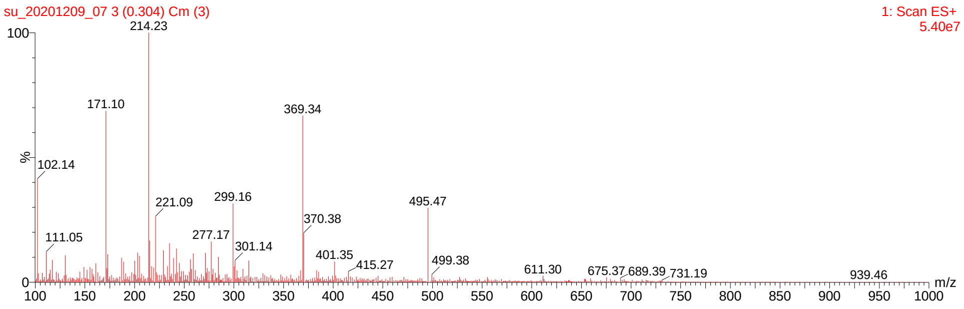

Supplementary Figure 6. Mass spectrum of **1**.

<u>Synthesis of **2**</u>

To a solution of **1** (51.6 mg, 0.11 mmol) in THF (1.5 mL) and water (0.5 mL) was added potassium hydroxide (KOH) (12.2 mg, 0.22 mmol). After stirring at room temperature for 1 h, 1 N HCl aq. was added, and the mixture was extracted with ethyl acetate. The organic layer was dried over sodium sulfate, filtered, and evaporated to give **2** as a white solid (27.3 mg, 56% yield). ESI-MS (m/z): calcd. for $C_{29}H_{47}O_3$, 443; found 443, $[M–H]^–$.

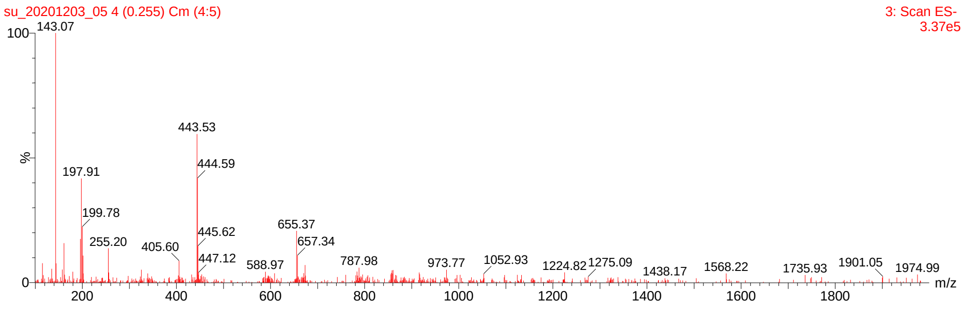

Supplementary Figure 7. Mass spectrum of **2**.

<u>Synthesis of Bio–PEG3–cholesterol</u>

A solution of **2** (13.3 mg, 0.03 mmol), Biotin–PEG3–amine (13.8 mg, 0.03 mmol), 1-[bis(dimethylamino)methylene]-1*H*-1,2,3-triazolo[4,5-b]pyridinium 3-oxide hexafluoro-phosphate (HATU) (17.1 mg, 0.05 mmol) and TEA (8.3 μL, 0.06 mmol) in 1 mL of anhydrous DMF was stirred overnight at room temperature under an argon atmosphere. Brine:water (1:1) was added, and extracted with ethyl acetate twice. The combined organic layers were dried over sodium sulfate, filtered, and evaporated. The residue was purified by flash silica gel column chromatography (dichloromethane:methanol 15:1) to yield Bio–PEG3–cholesterol (22.9 mg). The Bio–PEG3–cholesterol (7.7 mg) was further purified by

semipreparative HPLC using eluent A (H_2O with 0.1% TFA) and eluent B (methanol) (A:B = 90:10–0:100 for 20 min then 0:100 for 40 min) to yield Bio–PEG3–cholesterol as a white solid (5.3 mg). ^{1}H NMR (400 MHz, DMSO-d_6): δ 7.84 (t, J = 5.6 Hz, 1 H), 7.57 (t, J = 5.4 Hz, 1 H), 6.43 (s, 1 H), 6.36 (s, 1 H), 5.33 (m, 1 H), 4.30 (m, 1 H), 4.13 (m, 1 H), 3.87 (s, 2 H), 3.50 (br.s., 8 H), 3.44–3.37 (6 H), 3.27–3.16 (8 H), 2.81 (dd, J = 12.4 Hz, 4.8 Hz, 1 H), 2.57 (d, J = 12.8 Hz, 1 H), 2.35 (m, 1 H), 2.16 (m, 1 H), 1.98–1.79 (m, 4 H), 1.65–0.96 (m, 29 H), 0.90–0.83 (m, 9 H), 0.65 (s, 3 H); ESI-MS (m/z): calcd. for $C_{47}H_{81}N_4O_7S$, 846; found 846, $[M+H]^+$.

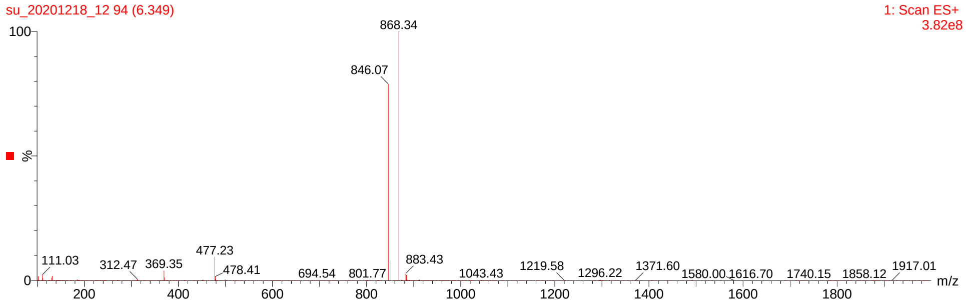

Supplementary Figure 8. Mass spectrum of Bio–PEG3–cholesterol.
